# Gaseous Ammonia Sensing from Liquids via a Portable Chemosensor with Signal Correction for Humidity

**DOI:** 10.3390/bios15070407

**Published:** 2025-06-25

**Authors:** Andrea Rescalli, Ilaria Porello, Pietro Cerveri, Francesco Cellesi

**Affiliations:** 1 Department of Chemistry, Materials and Chemical Engineering “G. Natta”, Politecnico di Milano, 20133 Milan, Italy; andrea.rescalli@polimi.it (A.R.); ilaria.porello@polimi.it (I.P.); 2 Department of Electronics, Information and Bioengineering, Politecnico di Milano, 20133 Milan, Italy; 3 Department of Industrial and Information Engineering, Università degli Studi di Pavia, 27100 Pavia, Italy; pietro.cerveri@unipv.it

**Keywords:** ammonia sensing, polyaniline, chemosensors, point-of-care testing

## Abstract

Ammonia (NH_3_) detection in liquids and biological fluids is essential for monitoring environmental contamination and industrial processes, ensuring food safety, and diagnosing health conditions. Existing detection techniques are often unsuitable for point-of-care (POC) use due to limitations including complex sample handling, lack of portability, and poor compatibility with miniaturized systems. This study introduces a proof-of-concept for a compact, portable device tailored for POC detection of gaseous ammonia released from liquid samples. The device combines a polyaniline (PANI)-based chemoresistive sensor with interdigitated electrodes and a resistance readout circuit, enclosed in a gas-permeable hydrophobic membrane that permits ammonia in the vapor phase only to reach the sensing layer, ensuring selectivity and protection from liquid interference. The ink formulation was optimized. PANI nanoparticle suspension exhibited a monomodal, narrow particle size distribution with an average size of 120 nm and no evidence of larger aggregates. A key advancement of this device is its ability to limit the impact of water vapor, a known source of interference in PANI-based sensors, while maintaining a simple sensor design. A tailored signal processing strategy was implemented, extracting the slope of resistance variation over time as a robust metric for ammonia quantification. The sensor demonstrated reliable performance across a concentration range of 1.7 to 170 ppm with strong logarithmic correlation (R^2^ = 0.99), and very good linear correlations in low (R^2^ = 0.96) and high (R^2^ = 0.97) subranges. These findings validate the feasibility of this POC platform for sensitive, selective, and practical ammonia detection in clinical and environmental applications.

## 1. Introduction

Detecting ammonia in liquids and biological fluids is critical for a wide range of applications, including the monitoring of environmental pollution [1,2], quality control in industrial and food production processes [3,4], and the early diagnosis of certain medical conditions [5,6]. Accurate and timely measurement of ammonia levels can help prevent harmful exposure, ensure regulatory compliance, and support effective clinical decision-making [7,8].

Ammonia is physiologically present in body fluids such as blood, plasma, and urine as a result of amino acid metabolism [9,10]. Elevated concentrations of ammonia are a strong indicator of pathological nitrogen homeostasis and are associated with serious conditions such as chronic kidney disease, urinary tract obstruction, gastrointestinal bleeding, peptic ulcers, and recently COVID-19 [11,12,13,14,15,16,17]. At excessive levels, ammonia is neurotoxic and can lead to encephalopathy [18]. Hence, accurate and timely detection of ammonia concentrations can result in early diagnostics of these life-threatening diseases [19].

Existing methods for diagnosing and managing ammonia-related conditions are time-consuming and complex. The need for specialized equipment, as well as sample pre-treatment procedures and careful handling to avoid contamination and alterations in ammonia concentrations, complicate the diagnostic process [20]. Rapid, cost-effective, and easy-to-use techniques for measuring ammonia levels in blood and urine are of utmost importance. Point-of-care (POC) testing presents a promising alternative to tackle these issues and improve diagnostic efficiency [21]. Chemoresistive sensors are gaining huge interest due to their low cost, simplicity, and scalability. Conducting polymers in particular, such as polyaniline (PANI), have become essential in gas sensing thanks to their advantages such as ease of fabrication, design flexibility, and ability to detect gases at ambient temperature [22,23,24,25]. Traditional PANI-based sensors exploit the redox and protonation properties of the polymer, where exposure to NH_3_ leads to deprotonation of the conductive emeraldine salt form, resulting in a measurable increase in resistance. However, pristine PANI sensors are known to suffer from key limitations, particularly in terms of selectivity and sensitivity under varying environmental conditions, such as humidity [26]. Recent advances have addressed some of these limitations through the development of PANI-based composites, where the polymer is integrated with nanostructured or functional materials. For instance, high-performance sensors have been demonstrated using PANI/Nb_2_CT_x_ nanosheets [27,28,29], PANI/SrGe_4_O_9_ nanocomposites [30], and PANI/halloysite nanotubes [31]. These materials enhance sensitivity and selectivity through increased surface area, tailored adsorption sites, or heterogeneous interface interactions. In addition, such composite materials can mitigate the negative impact of humidity on sensor performance. While such approaches have shown promise, they often involve complex fabrication steps, multicomponent synthesis, or reduced compatibility with low-cost, scalable production.

**Figure 1:**
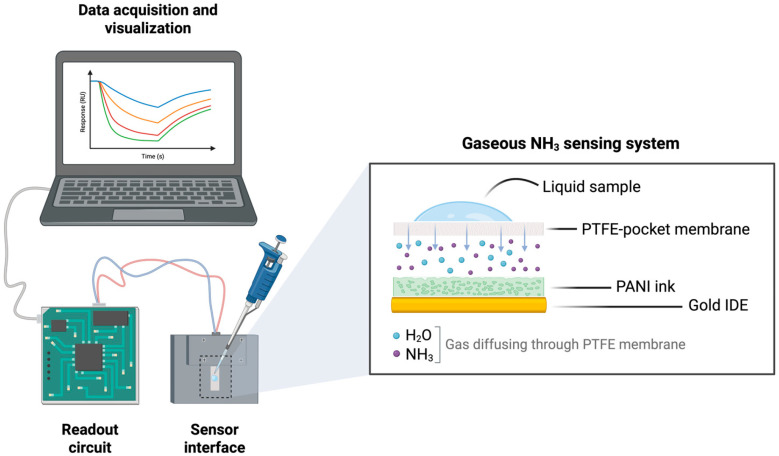
Schematic representation of the developed device. A sensor interface is connected to a readout acquisition circuit, and the data are transmitted to a host computer where a dedicated graphical user interface enables interactions with the device. A zoomed-in view of the structure of the chemosensor shows the PANI ink layer deposited onto the gold surface of the IDE used as transducer. The PTFE pocket-membrane envelops the chemosensor and enables diffusion of analytes in gaseous phase through its pores, so that the liquid sample cannot directly interact with the polymeric layer. This figure was created with BioRender.com.

In this study, we developed a proof-of-concept of a miniature, portable device suitable for point-of-care detection of gaseous ammonia from liquid samples, compatible with the analysis of biological fluids (Figure 1). This device integrates accurate resistance measurements to track variations in the conductivity of PANI-based chemosensors. Rather than introducing material-level modifications, our focus was to preserve a simple sensor architecture. Modifications to the previously reported PANI ink formulation protocols [22,32] have been introduced to guarantee a better deposition process. A gas-permeable hydrophobic membrane was used to physically isolate the sensing film from liquid contact, while ad hoc signal processing strategies were developed to limit water interference and properly correlate the sensor response with ammonia concentration. This system-level strategy enables effective sensing despite the known limitations of pristine PANI films and supports the development of robust and accessible POC devices.

## 2. Materials and Methods

### 2.1. Materials and Reagents

Dodecylbenzene sulfonic acid (soft type, mixture) > 95% (DBSA) was purchased from Tokyo Chemical Industry (TCI, Zwijndrecht, Belgium). Ethanol (absolute anhydrous) 99.9% was purchased from Carlo Erba Reagents (Cornaredo, Italy). Ammonium persulfate reagent grade 98% (APS), Aniline (MW = 94.13 g/mol) > 99.5%, Sodium dodecyl sulfate > 98.5% (SDS), and the polytetrafluoroethylene filter discs (PTFE, 0.2 μm pore size) were purchased from Sigma-Aldrich (Merck Life Science, Milan, Italy). All chemicals were used without further purification unless otherwise indicated. Deionized water (18.2 MΩ) was obtained from a Millipore Milli-Q purification unit, and 100 μm-spaced interdigitated electrodes (IDEs) were purchased from Metrohm Italiana Srl (Milan, Italy).

### 2.2. PANI Ink Formulation

**Figure 2:**
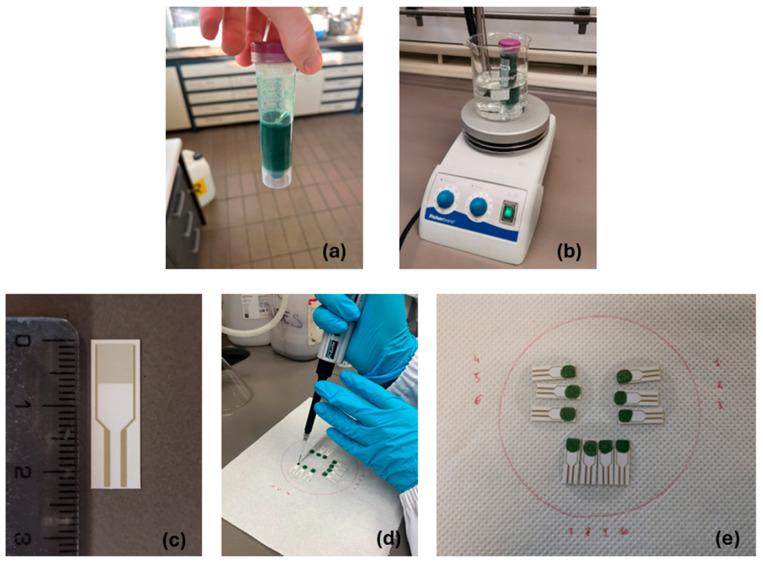
Steps of the PANI ink preparation and chemosensor fabrication. (**a**) Synthesized PANI ink. (**b**) Heating of the PANI ink before deposition. (**c**) Details of the IDE geometry. The overall dimensions of the IDE are 7.6 × 22 × 0.7 mm, with 100 µm spacing. (**d**) PANI ink drop-casting onto the transducers. (**e**) Chemosensors ready for use.

PANI colloidal dispersion was obtained via a modified rapid mixing method [22,32]. DBSA was mixed with Milli-Q water to obtain a 0.09 g/mL solution (*Sol. A*), then 20 mL of *Sol. A* was used to dissolve 0.36 g of APS (*Sol. B*). A total of 0.6 mL of aniline was added dropwise to 20 mL of *Sol. A,* maintaining the system under stirring (200 RPM) at room temperature until complete dissolution was reached, avoiding foam formation (*Sol. C*). *Sol. B* was quickly dropped into *Sol. C,* stirring the resulting dispersion for 3 h (250 RPM) at 25 °C (*Sol. D*). A total of 26.1 g of SDS was dissolved in 1.8 L of Milli-Q water (0.05 M, *Sol. E*). A total of 20 mL of *Sol. E* was added to *Sol. D* stirring the mixture until homogeneity was reached. The obtained mixture was ultra-centrifuged for 30 min at 6000 RPM. The collected supernatant was inserted into a 14 kDA MWCO dialysis bag and dialyzed against 500 mL of *Sol. E.* The external solution was changed three times for an overall dialysis time of 48 h. The synthesized PANI ink (Figure 2a) was then stored at 4 °C in a dark environment.

The particle size distribution of PANI nanoparticles was evaluated via Dynamic Light Scattering (DLS) using a Malvern Zetasizer Nano ZS, equipped with a 4 mW He−Ne laser operating at λ = 634 nm (backscattered angle 173°). A total of 0.5 mL of PANI ink was diluted with 4.5 mL of Milli-Q water prior to DLS analysis. Ultraviolet–visible (UV-Vis) absorption spectrum of PANI nanoparticle suspension (0.1 mg/mL) was obtained using a Hewlett Packard 8453 UV-Vis spectrophotometer (Agilent Technologies, Cernusco sul Naviglio, Italy).

### 2.3. Chemosensor Fabrication and Device Integration

#### 2.3.1. Ink Deposition

Electrodes were immersed for 30 min in a 1:1 solution of ethanol and deionized water for cleaning. Each electrode was then dried using a nitrogen flux. The PANI ink stored at 4 °C was gradually heated up to 25 °C (Figure 2b), then 10 μL of ink was manually drop-casted at the center of each cleaned IDE (Figure 2c,d). As a final step, the chemosensors were left to dry for 1 h under a diffused nitrogen flux. After a 1.5 h resting period in a closed environment, the functionalized chemosensors were ready for image analysis and resistance measurements (Figure 2e). Image analysis was carried out using a LEXT OLS4100 3D laser scanning confocal microscope from Olympus Corporation (Segrate, Italy). The conductivity of six sensors was measured for 3 min immediately after preparation. Afterwards, these electrodes were then divided into two groups: three were stored at room temperature in the dark, while the other three were kept at 4 °C under the same conditions. Baseline resistance was measured on days 1, 2, 3, 4, and 7, and normalized to the initial baseline recorded on day 0.

#### 2.3.2. Integration of Porous Membrane

A custom pocket was fabricated using a gas-permeable, hydrophobic PTFE membrane filter. The pocket integrates the hydrophobic membrane into the upper wall of a small, sealed plastic pouch. The sensor is inserted between the two walls of the pocket, positioned directly beneath the membrane. When a liquid sample is deposited on the external surface of the membrane, only vapor from the sample permeates through the membrane and diffuses into the enclosed volume, where it can interact with the sensor surface. This configuration provides a protected environment for the sensor, preventing deterioration due to direct contact with the liquid while enabling gas-phase detection of the target analyte.

#### 2.3.3. Portable Device

To meet the goal of developing a small, portable device, an electronic platform based on a CY8C5888LTI-LP097 microcontroller unit was developed to measure the chemosensor response. The resistance of each sensor was determined using a reference resistor method in a four-wire configuration by introducing a small, non-disruptive current through the sensor. An excitation current of 10 µA was selected as a compromise between the accuracy of the measurements and polarization and self-heating effects. A graphical user interface was developed with Python (ver. 3.9.11) using PyQt5 (ver. 5.15.2) as the main graphical framework to handle communication with the device, including data visualization and data export options.

### 2.4. Experimental Protocol and Data Pre-Processing

A single measurement lasted approximately 20 min, subdivided as follows:

(a) The chemosensor was encapsulated within the pocket-membrane and connected to the measuring device. A 3 min baseline acquisition period was initiated to establish the sensor’s initial resistance. The mean value of the final 30 s, termed $R_{b}$, served as the reference value for normalizing all subsequent resistance data;
(b) A 50 μL liquid sample containing varying concentrations of ammonia was dropped in the center of the pocket-membrane. The chemosensor was subsequently exposed to the resulting gaseous ammonia for 10 min;
(c) Following the exposure phase, the pocket-membrane was removed and the sensor re-exposed to ambient air for an additional 10 min to monitor its recovery characteristics.

The raw resistance data collected were normalized relative to $R_{b}$. A Savitzky–Golay filter, employing a polynomial order of 3 and a window size of 111, was applied to the normalized data. This process preserved the intrinsic morphology of the signal while effectively smoothing out noise.

## 3. Results and Discussion

### 3.1. Chemosensor Assembly and Characterization

PANI nanoparticles were synthesized via a modified rapid mixing method, based on the oxidative polymerization of aniline within a microemulsion system. The process involved the use of APS as an oxidizing agent under acidic conditions and SDS as a surfactant to provide colloidal stability. Compared to other formulation protocols reported in the literature, the reaction time between aniline and APS was intentionally extended to promote a more uniform conversion. In fact, following the procedures reported in previously published protocols [22,32], visual inspection revealed incomplete polymerization, with residual white areas present instead of a uniform dark green hue. Extending the reaction time to 3 h ensured a consistent appearance throughout the sample. Additionally, both the duration and speed (30 min at 6000 RPM) of the centrifugation step were increased, which significantly improved the texture of the colloidal dispersion, resulting in a more uniform and less viscous mixture. Finally, purification through regular replacement of the external solution during dialysis further improved the quality of the dispersion by effectively removing unreacted components and byproducts.

**Figure 3:**
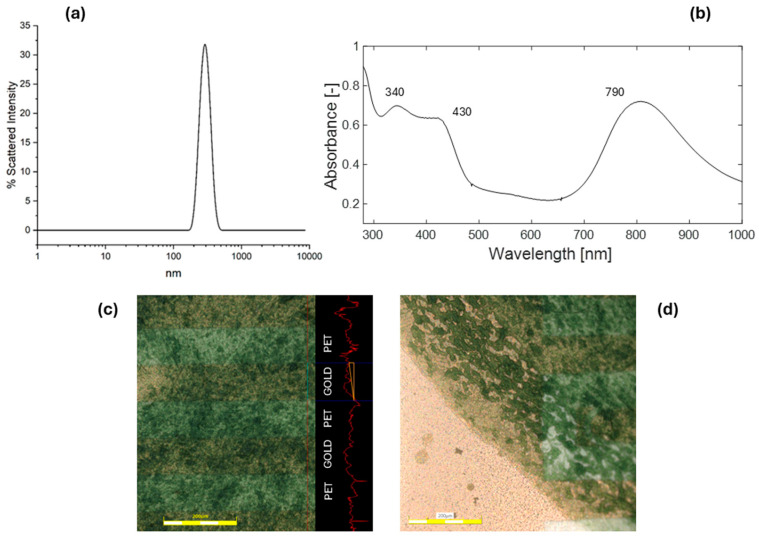
Characterization of the PANI nanoparticle dispersion and ink deposition. (**a**) Size distribution curve of the PANI nanoparticle dispersion, obtained by DLS. (**b**) UV-Vis spectrum of the PANI ink. (**c**) Optical imaging and profilometry of the central region of the polyaniline film. (**d**) Optical imaging of the edge of the polyaniline film. Scale bar of c, d = 200 μm.

The DLS analysis of the PANI nanoparticle suspension revealed a monomodal, narrow particle size distribution with an average size of 120 nm, with no evidence of larger aggregates (Figure 3a). The UV–Vis analysis of the suspension exhibits characteristic absorption bands indicative of conductive PANI in its emeraldine salt form (Figure 3b). Specifically, peaks are observed at 340 nm, attributed to π–π* transitions in benzene rings; at 430 nm, corresponding to exciton-coupled π*–polaron transitions; and around 790 nm, associated with π–polaron transitions [33,34].

The improvement of the physicochemical properties of the PANI ink not only facilitated deposition but also enhanced the uniformity of the resulting film in both thickness and composition. A detailed examination with laser scanning confocal microscopy of the central region of the functionalized electrodes confirmed that the film thickness remained consistent, despite the irregular height profile caused by the alternating gold and plastic bands of the IDEs (Figure 3c). Nonetheless, the film exhibited more aggregated regions at the edges, along with a less dense boundary band. These features are characteristic of the drying process and are associated with the manual drop-casting deposition procedure (Figure 3d).

**Figure 4:**
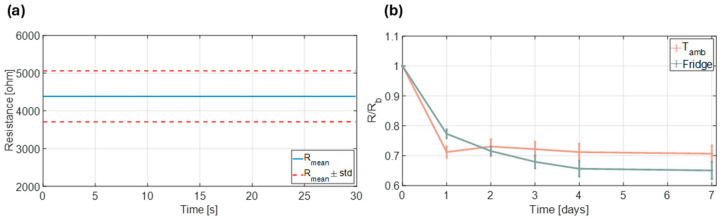
Characterization of the fabricated chemosensors. (**a**) Average of the last 30 s of baseline resistance of a batch of 6 electrodes after production, at ambient temperature. (**b**) Chemosensors’ loss of performance in terms of normalized baseline resistance with respect to storage conditions (ambient temperature or fridge).

Variations in drying patterns and surface coverage were expected to influence each chemosensor’s baseline resistance. To explore this, a batch of six sensors was prepared and their conductivity monitored over a 3 min period. By averaging the last 30 s of each measurement, a mean resistance of 4.38 kΩ (SD: 0.674 kΩ) was computed, indicating a ±15% deviation from the overall batch baseline (Figure 4a). In addition, the effect of storage conditions on the sensor conductivity was investigated. The six electrodes were divided into two groups: three were stored at room temperature in a dark environment, and the other three were maintained at 4 °C under identical conditions. Baseline resistance was measured daily on days 1 through 7, then normalized to the initial baseline recorded immediately after production (day 0). Sensors kept at room temperature exhibited a 30% drop in baseline resistance from day 1, but then remained stable throughout the week. In contrast, sensors stored at 4 °C exhibited an exponential decay pattern, with a lower initial loss after 1 day (20%) and a more pronounced decline (35%) after one week (Figure 4b). Despite the refrigerated sensors undergoing greater overall decay, the more controlled and predictable loss suggests that storage at 4 °C may be the preferable condition.

### 3.2. Exploring Operational Range—NH_3_ Sensing

**Figure 5:**
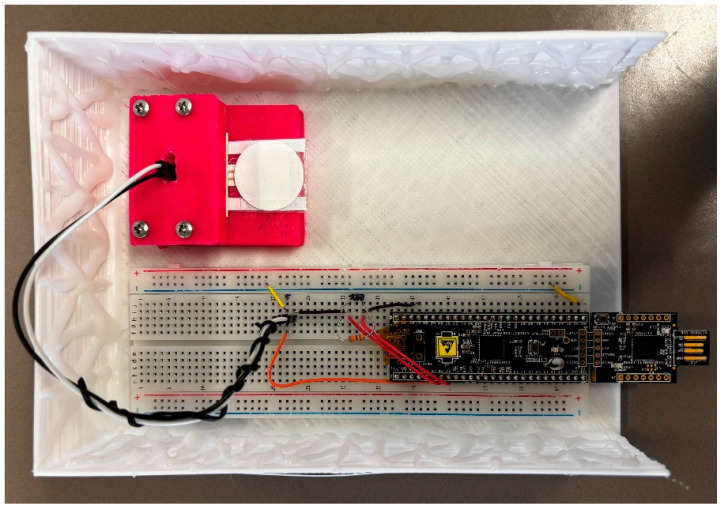
Top view of the portable device with the chemosensor enveloped in the pocket-membrane.

The dimensions of the developed device were compatible with a POC scenario (Figure 5). The reference resistor method adopted during the measurement phase was optimized to (a) mitigate measurement errors induced by wire resistances; (b) quantify and subtract from the measurement the overall offset error introduced by the signal acquisition chain; and (c) cancel out gain errors of the analog-to-digital converter and the current generator used as excitation source. The developed system showed excellent performance in the 0.1 to 10 kΩ range, with a maximum percentage accuracy error lower than 2% when compared to a commercially available digital multimeter used as a reference.

**Figure 6:**
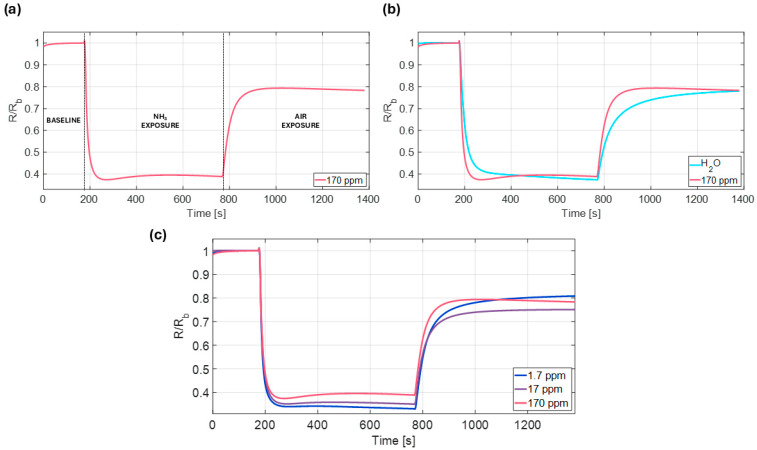
Chemosensor response when exposed to a liquid sample. (**a**) Using exposure to a liquid sample containing 170 ppm of NH_3_ as a representative case, the three phases of the protocol are highlighted: baseline acquisition, NH_3_ exposure, and air exposure. (**b**) Comparison between the response elicited by exposure to NH_3_ and exposure to water alone. (**c**) Comparison between the responses elicited by exposure to NH_3_ at different concentrations.

Upon deposition of a liquid sample onto the pocket-membrane, a significant drop in resistance occurred due to the interaction of water vapor with the PANI ink [26]. In fact, water molecules contribute to the protonation effects of this conductive polymer, thus increasing its conductivity [35]. This initial interference delayed the onset of the ammonia response, eventually resulting in an increase in sensor resistance, as observed in the positive deflection in the central part of the curve (Figure 6a). Once the pocket-membrane, along with the sample, was removed, the sensor gradually began to recover towards its initial state, although it was unable to fully return to its original condition (Figure 6a). This behavior can be attributed to multiple mechanisms. The deprotonation of the PANI film by ammonia involves the adsorption of NH_3_ molecules onto the polymer surface. The adsorption and desorption kinetics are not symmetric [36], and some ammonia may diffuse within the polymer matrix, contributing to delayed or incomplete baseline recovery [37]. At the same time, water vapor interference induces protonation of the polymer backbone, which is responsible for the sudden drop in resistance. It has been shown that protonation and deprotonation can induce permanent changes in the surface morphology of PANI films, and that multiple cycles are typically required to reach a stable, equilibrium state [38]. These morphological rearrangements inevitably alter the electrical characteristics of the material. In our case, such effects result in an incomplete return to baseline after desorption, which we consider acceptable given the current intended use of the sensor. The device is designed as a POC platform for liquid sample screening, where single-use or non-continuous operation is performed. Therefore, the irreversible changes observed after exposure do not affect measurement accuracy, as no consecutive measurements are performed on the same sensor.

Quantifying the distinct features of the chemosensor response curve was crucial to reliably correlating the ammonia concentration in the sample with the analytical signal obtained. As previously indicated, water vapor represents a major interfering species in this experimental configuration. To mitigate its contribution and accurately isolate the ammonia-related signal, a dedicated analytical method was developed. Focusing on the central part of the response curve, two key indicators were identified:

The minimum of the $R/R_{b}$ ratio reached right after the sample drop;

The local maximum of the $R/R_{b}$ ratio reached after the sample drop.

From these two indicators, the *slope* parameter was computed, defined as:

(1) $$Slope=\frac{max\left(R/R_{b}\right)-min\left(R/R_{b}\right)}{t_{max}-t_{min}}$$

where $t_{max}$ and $t_{min}$ represent the time at which the maximum and minimum values are reached, respectively. This parameter was fundamental in constructing a calibration curve, as it remained insensitive to water vapor. Only ammonia-containing samples were in fact characterized by a maximum following the minimum in the response (Figure 6b). The reason is attributed to the role of NH_3_ as a base, which deprotonates the emeraldine salt form of the PANI film. This attained deprotonation of the -NH groups in the polymer backbone results in a reduction in its conductivity, thereby increasing its resistance [39,40,41]. Ammonia sensors based on PANI ink films typically exhibit responses to NH_3_ concentrations ranging from a few to hundreds ppm [22,26,42,43]. In this work, liquid samples containing NH_3_ ranging from 1.7 to 170 ppm were tested using the setup described earlier (Figure 6c).

**Figure 7:**
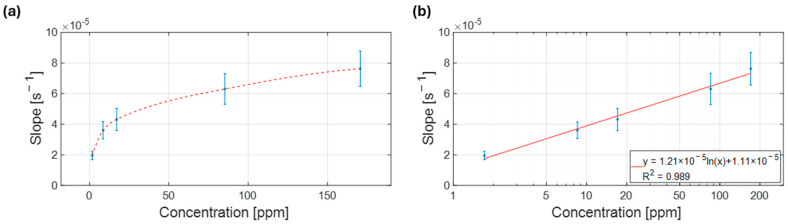
Calibration curves relating the response of the chemosensors, in terms of slope, to the concentration of NH_3_ in the sample. (**a**) Full range of concentrations tested, from 1.7 to 170 ppm. (**b**) Full range calibration curve on the logarithm of concentrations.

Sensors were fabricated and exposed to ammonia concentrations from 1.7 to 170 ppm. The response slope was calculated using Equation (1). When plotting the slope of each observation against the ammonia concentration in the sample, a logarithmic trend emerged (Figure 7a). At lower concentrations, the chemosensors demonstrated enhanced sensitivity; however, at higher concentrations, saturation of the interaction sites between NH_3_ molecules and the conducting polymer layer led to a more attenuated response. In fact, beyond a certain concentration, the PANI film is affected by proton deficiency, and its conductivity cannot be further modulated by the presence of reducing agents [40]. The slope parameter was hence plotted against the natural logarithm of the concentration, revealing a strong linear correlation (R^2^ = 0.99, Figure 7b).

**Figure 8:**
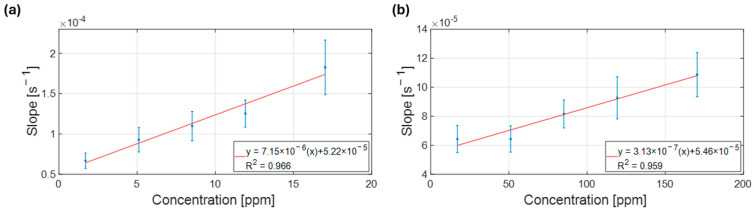
Calibration curves relating the response of the chemosensors, in terms of slope, to the concentration of NH_3_ in the sample. (**a**) Linear lower concentration range, calibration curve from 1.7 to 17 ppm. (**b**) Linear higher concentration range, calibration curve from 17 to 170 ppm.

Based on these findings, two additional calibration curves were constructed to separately address the low and high concentration ranges. In the low range (1.7–17 ppm), a linear correlation of the slope with concentration was detected (R^2^ = 0.97, Figure 8a). Similarly, a linear correlation was maintained in the higher concentration range (17–170 ppm) with an R^2^ value of 0.96 (Figure 8b).

## 4. Conclusions

In this study, we developed a robust, portable platform that seamlessly integrates an accurate resistance readout circuitry and a chemoresistive sensor based on a conducting polymer to measure the amount of gaseous ammonia from liquid samples. Interdigitated electrodes served as transducers, while a drop-cast film of PANI ink worked as the selective recognition layer. With respect to the literature, the protocol for the synthesis of the ink was modified to promote a more homogeneous conversion reaction. This adjustment yielded a narrowly sized distributed colloidal dispersion, thus improving the deposition process. A custom pocket was fabricated using a gas-permeable, hydrophobic membrane filter to envelop the sensor, preventing direct contact with liquid samples and ensuring that only the vapor phase interacts with the PANI ink film. To mitigate water vapor interference, which represents a well-known limitation in PANI-based sensors, a specific signal processing strategy was carried out to extract the slope of the sensor resistance variation upon exposure to ammonia. This approach effectively isolated the ammonia response from water-induced fluctuations, ensuring more reliable measurements. Concentrations from 1.7 to 170 ppm were tested, and a logarithmic correlation with the slope parameter was detected (R^2^ = 0.99). The full range was split into low (1.7 to 17 ppm) and high (17 to 170 ppm) concentration intervals, linearly correlated with the slope parameter (R^2^ = 0.97 and R^2^ = 0.96, respectively).

Overall, these results confirm the feasibility of our portable, chemosensor-based device for the POC detection of ammonia concentrations from biological fluids, and, in conclusion, this technology holds significant promise for early diagnostics of life-threatening diseases. Nevertheless, some limitations remain. In particular, a more comprehensive characterization of the sensor performance, including recovery times and long-term stability, requires further investigation. pH and temperature can affect the NH_3_/NH_4_^+^ equilibrium in solution, and consequently the headspace concentration. Future work may involve a detailed quantification of their influence on sensor performance. Alternative IDE geometries may also be explored to potentially enhance sensitivity. These efforts will build on the preliminary results presented here to further validate and optimize the proposed sensing system. Finally, as PANI is known to exhibit cross-sensitivity to a wide range of volatile and semi-volatile compounds, including electron acceptors, donors, and organic vapors [37,44,45], its use in complex biological matrices poses inherent challenges in terms of selectivity. In this initial study, we prioritized simplicity in sensor design, focusing on mitigating water vapor interference through a dedicated signal processing strategy. Further investigation may focus on the sensor response to interferents and potential methods to enhance selectivity, such as material composites or sensor arrays.

## Data Availability

Data is contained within the article.

## Abbreviations

The following abbreviations are used in this manuscript:

APS	Ammonium persulfate
DBSA	Dodecylbenzene sulfonic acid
DLS	Dynamic Light Scattering
IDE	Interdigitated electrode
NH_3_	Ammonia
PANI	Polyaniline
PTFE	Polytetrafluoroethylene
POC	Point-of-care
SDS	Sodium dodecyl sulfate
